# Percutaneous mechanical thrombectomy of superior mesenteric artery embolism

**DOI:** 10.2478/raon-2013-0029

**Published:** 2013-07-30

**Authors:** Dimitrij Kuhelj, Pavel Kavcic, Peter Popovic

**Affiliations:** Clinical Radiology Institute, University Medical Centre Ljubljana, Ljubljana, Slovenia

**Keywords:** acute mesenteric ischemia, superior mesenteric artery embolism, percutaneous mechanical thrombectomy, Aspirex^®^, Rotarex^®^

## Abstract

**Background:**

The present series present three consecutive cases of successful percutaneous mechanical embolectomy in acute superior mesenteric artery ischemia. Superior mesenteric artery embolism is a rare abdominal emergency that commonly leads to bowel infarction and has a very high mortality rate. Prompt recognition and treatment are crucial for successful outcome. Endovascular therapeutic approach in patients with acute SMA embolism in median portion of its stem is proposed.

**Case reports.:**

Three male patients had experienced a sudden abdominal pain and acute superior mesenteric artery embolism in median portion of its stem was revealed on computed tomography angiography. No signs of intestinal infarction were present. The decision for endovascular treatment was made in concordance with the surgeons. In one patient 6 French gauge Rotarex^®^ device was used while in others 6 French gauge Aspirex^®^ device were used. All patients experienced sudden relief of pain after the procedure with no signs of intestinal infarction. Minor procedural complication – rupture of a smaller branch of SMA during Aspirex^®^ treatment was successfully managed by coiling while transient paralytic ileus presented in one patient resolved spontaneously. All three patients remained symptom-free with patent superior mesenteric artery during the follow-up period.

**Conclusions:**

Percutaneous mechanical thrombectomy seems to be a rapid and effective treatment of acute superior mesenteric artery embolism in median portion of its stem in absence of bowel necrosis. Follow-up of our patients showed excellent short- and long-term results.

## Introduction

Acute mesenteric ischemia (AMI) is a serious abdominal emergency characterized by sudden interruption of intestinal blood flow that commonly leads to bowel infarction. Its occurrence is relatively infrequent; accounting for 0.1% of hospital admissions.^1^ It typically affects elderly with increased risk of cardiovascular events. The most common cause of acute AMI is embolism (40–50% of cases).^2^ The majority of mesenteric emboli originate from the heart, most commonly in patients with atrial fibrillation.^3^

Despite considerable improvements in diagnostics and AMI treatment over the last decades, the condition still has a poor prognosis, with an in-hospital mortality rate of 59 to 93%.^4^ Early recognition and treatment are crucial for successful outcome.^4^ Delay in diagnosis results in intestinal infarction and gangrene that cannot be reversed by blood flow restoration. The treatment of choice has been surgical laparotomy with thrombectomy.^5^ Alternatively, cases of percutaneous thrombectomy were reported.^6^–^12^ The two main percutaneous methods are aspiration thrombectomy, in which thrombus is removed by suction, and mechanical thrombectomy, using different automated devices to fragment and remove embolus.

Aspirex S^®^ and Rotarex S^®^ (Straub Medical, Wangs, Switzerland) are mechanical thrombectomy devices that are often used in our department to treat acute and sub-acute peripheral arterial occlusions with good results and low complication rate. They are rotating over-the-wire devices designed for efficient and rapid removal of the occluding material. The rotations produce a continuous vacuum inside the catheter, which leads to aspiration of the material into the catheter and transportation into the collecting bag. Rotarex^®^ catheter features a rotating head detaching occlusion material in acute, subacute and chronic occlusions. Aspirex^®^ catheter is designed for the use in acute occlusions, since its head does not rotate, therefore the risk for vessel trauma should be lower as in Rotarex^®^ catheters. Both are 6 French gauge (Fr, F) and 8 Fr systems, suitable for the use in arteries with a diameter larger than 3 mm.^13^,^14^

The present series present three consecutive cases of successful percutaneous mechanical thrombectomy in acute superior mesenteric artery (SMA) embolism treated in our institution. Also, up to 45 months follow-up is presented.

## Case reports

**FIGURE 1.**
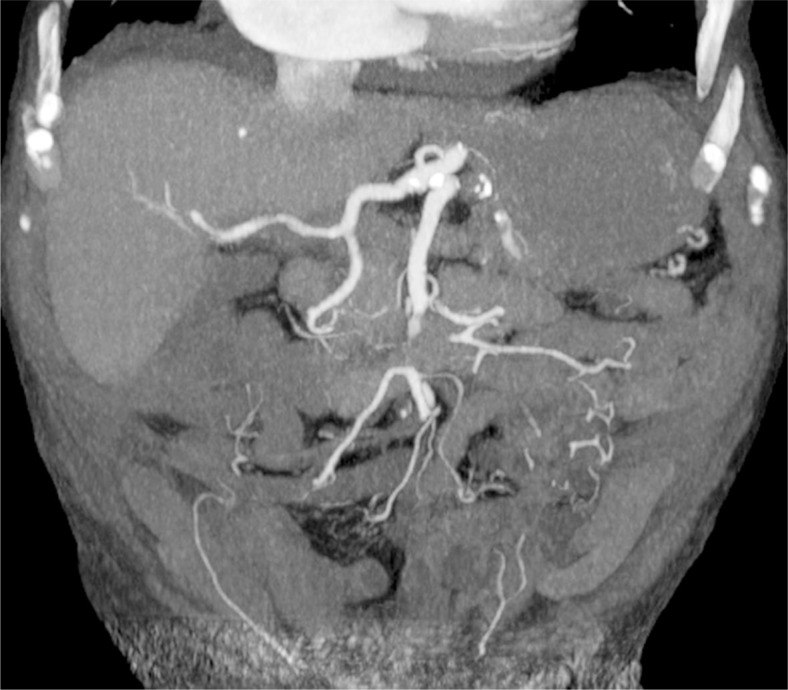
Coronal MIP reconstructions of CTA, revealing a segmental, occlusive acute embolism of the mid portion of SMA stem.

Three male patients, age 63–97 years, with sudden abdominal pain and also thoracic pain in one case were admitted to our hospital. Only one patient had known atrial fibrillation with congestive heart failure and was on Aspirin, while in two patients atrial fibrillation was newly discovered. Except for mild normocytic anaemia in one patient, no abnormal laboratory findings were present. Abdominal ischemia was suspected in two and dissection of thoracic aorta was suspected in patient with thoracic pain. Contrast enhanced computed tomography angiography (CTA) was performed accordingly. Segmental occlusion of the medial portion of SMA stem was revealed in all three patients. The occlusion of mid portion of SMA in one of our patients is shown on Figure 1. SMA proximally and distally from occlusion was of normal size in all three cases, suggesting embolism rather than thrombosis. No signs of irreversible bowel wall ischemia were found, though circumferential wall thickening of the cecum with normal, homogenous wall enhancement was noted in one patient.

The decision for endovascular treatment was reached in concordance with abdominal surgeons.

According to exiting angle of SMA from the aorta, transaxillary approach was used in two patients and transfemoral in one, local anaesthesia was used in all cases. Fifty-five cm long straight sheaths were used to catheterise SMA ostium. Initial SMA angiography confirmed acute occlusion in the mid portion of the SMA trunk with a few distal embolic occlusions in some jejunal and colic branches.

**FIGURE 2.**
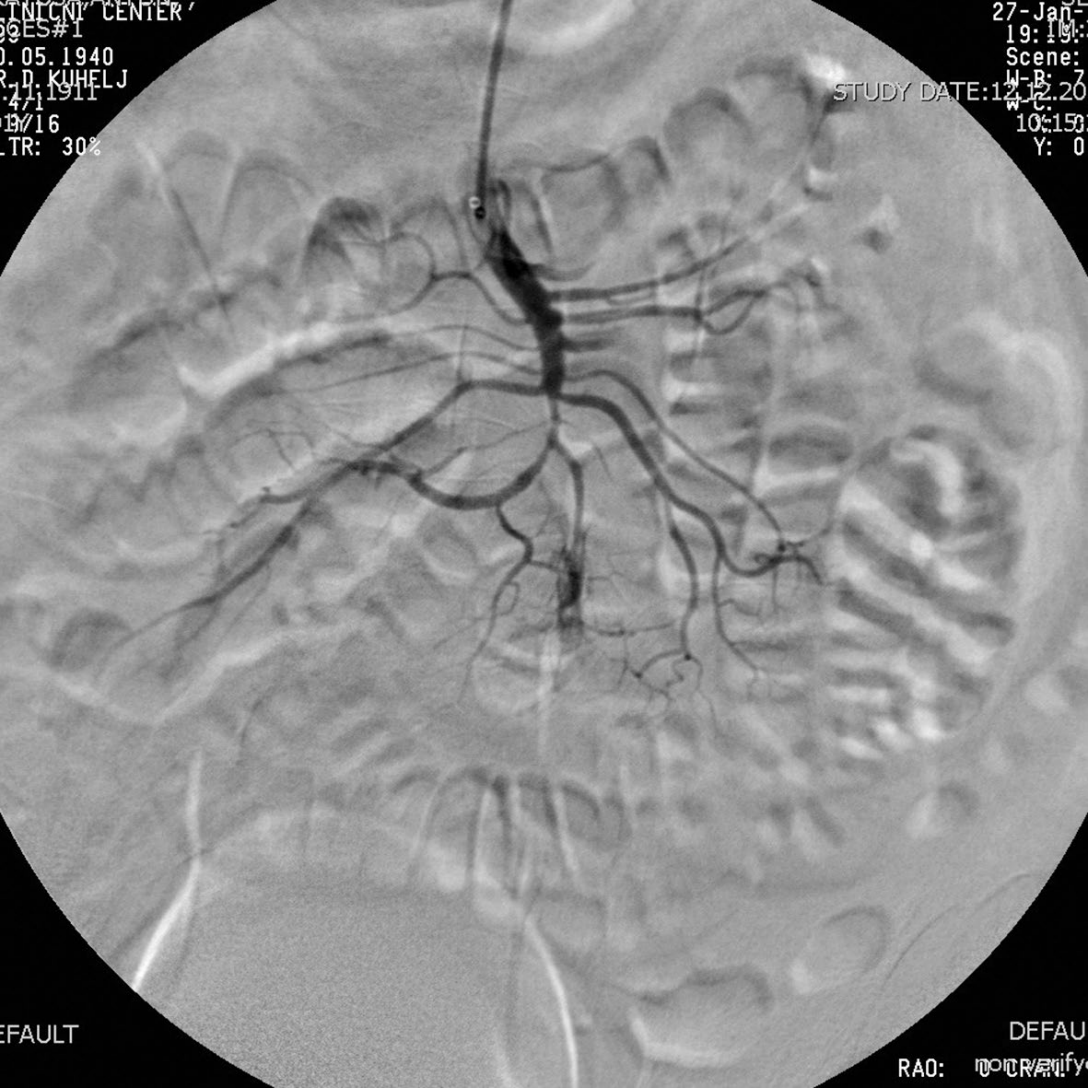
Control angiography after third pass with Aspirex^®^ showing patent SMA with extravasation of contrast medium due to a small branch rupture.

**FIGURE 3.**
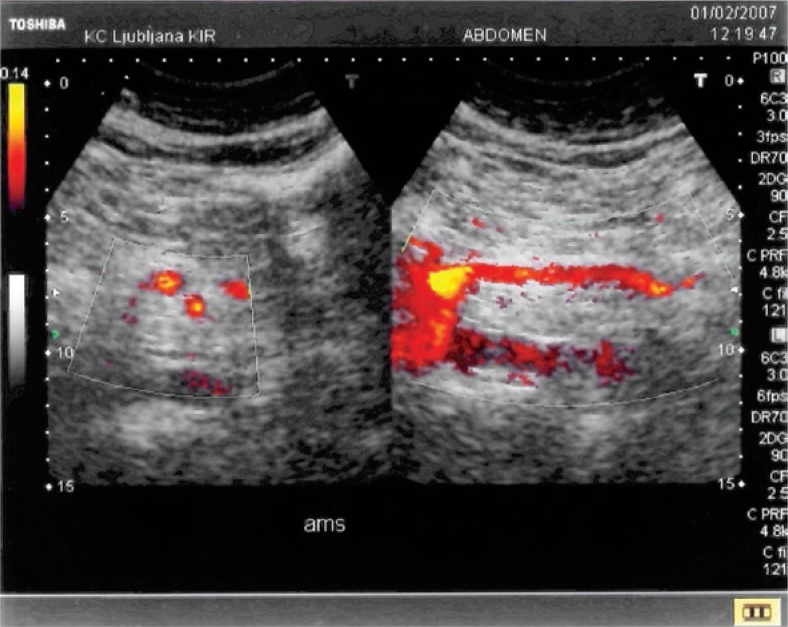
Control angiography after embolisation of ruptured branch with coils.

Six F Aspirex^®^ was used in two patients while in one case 6F Rotarex^®^ was used, since 6F Aspirex^®^ was not available at that time. Five thousand IU of heparin were infused intraarterially prior to the procedure in all cases. Two passes with catheter were performed in all patients, resulting in normal blood flow in the majority of peripheral branches with only minimal wall irregularity in SMA stem. During the third catheter manipulation in one patient, the guidewire slipped into the smaller branch and the third pass with the Aspirex^®^ ruptured it. The patient experienced acute pain and after angio-graphic confirmation of the rupture (Figure 2) embolization with coils sealed the rupture (Figure 3).

**FIGURE 4.**
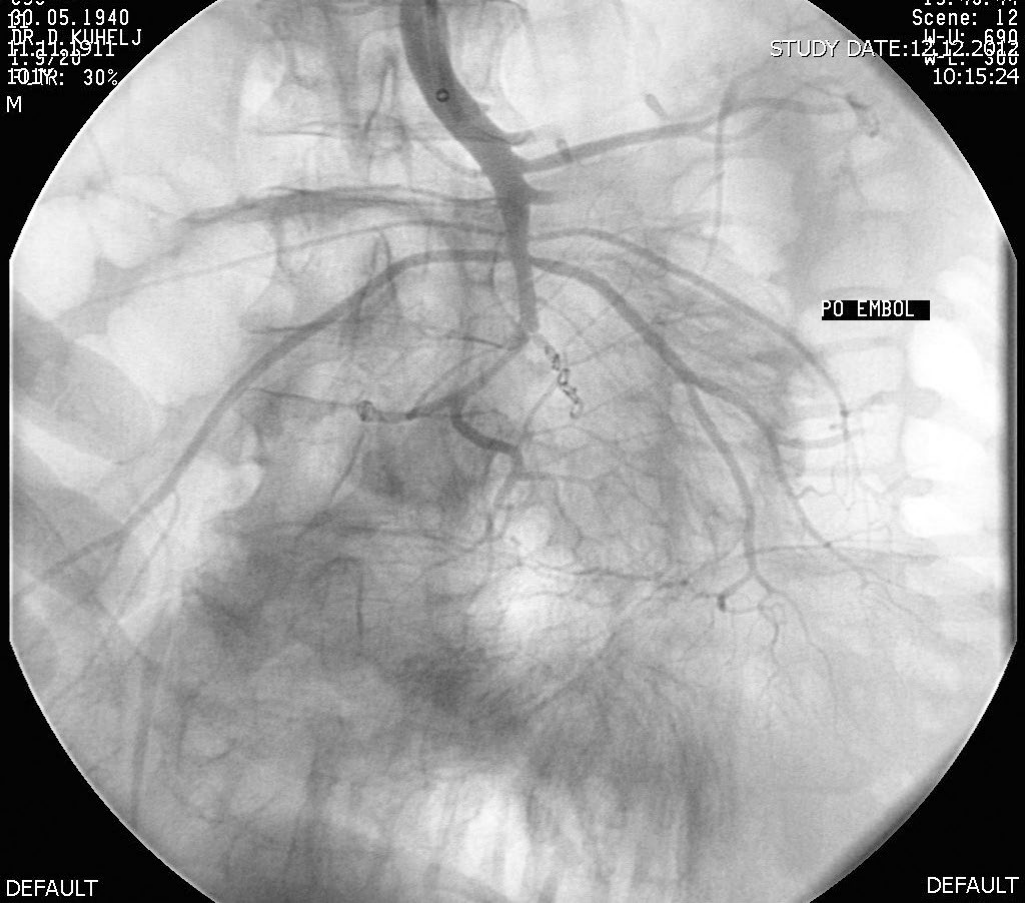
Doppler US control 5 days after the treatment showing patent SMA.

No adjunctive endovascular procedure was applied and the remaining distal embolic occlusions in jejunal and colic branches were left untreated. Patients’ symptoms improved immediately after the procedure in all cases. After a few days all patients underwent a colour Doppler ultrasound examination that confirmed patent SMA (Figure 4). In the patient with circumferential wall thickening of cecum control abdominal CTA due to distended and painful abdomen was performed also. A paralytic ileus of small bowel was found with no signs of bowel ischemia. SMA was patent with few non-occlusive wall thrombi. Ileus was treated conservatively and resolved spontaneously after 4 days. Lifelong warfarin therapy was introduced after discharge in two patients, while anaemia of unknown origin prevented it in one.

**FIGURE 5.**
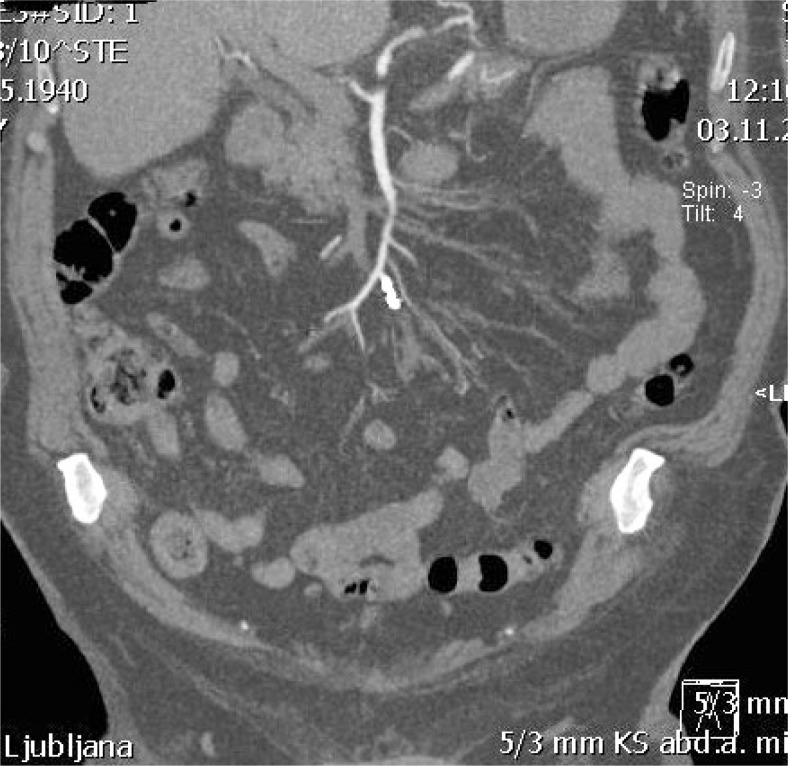
Coronal MIP reconstructions of CTA 45 months after the treatment, revealing patent SMA and branches. Coils permanently occluded ruptured branch.

Six-month follow-up excluded any clinical symptoms of abdominal ischemia in all patients and no follow-up was planned. 45 months after the treatment, the first patient treated had a CTA of abdominal and peripheral arteries prior to femoropopliteal bypass surgery. Normally patent SMA with no symptoms or signs consistent with recurrence of acute mesenteric ischemia was observed (Figure 5).

## Discussion

AMI is a life-threatening condition that commonly leads to bowel infarction. The reasons for the high mortality rate are late recognition due to non-specific clinical findings, infrequency of the condition as well as complex surgical treatment. The diagnosis of AMI depends on the ability of the attending physician to suspect and recognize it.^15^ In patients with acute abdominal pain and disproportionate lack of clinical, laboratory or ultrasound findings, AMI should be considered, especially if risk factors like atrial fibrillation, general atherosclerosis or hypercoagulability are present. Once AMI is suspected, CTA should be performed immediately in order to proceed with therapy as soon as possible. If there are clinical or CT signs of bowel necrosis, urgent surgery is needed. However, if there is no clear evidence of bowel necrosis, endovascular treatment can be a promising alternative^11^,^12^,^16^,^17^, as also confirmed in our series. Minimal bowel changes, such as circumferential wall thickening of cecum with normal, homogenous wall enhancement is not a contraidication for endovascular procedure. As follow-up showed also in our patient, there is no need for additional surgery, since the changes were due to oedema and not to prolonged, irreversible acute intestinal ischemia.^18^

To our knowledge, there are just few reports of using mechanical thrombectomy in SMA embolism treatment. One is the first case performed in our department^19^ and another is a case report of treatment with AngioJet^®^ hydrodynamic mechanical thrombectomy and EKOS^®^ catheter pharmacological thrombolysis.^12^ There are also some reports with a very high success rate (90%) for local fibrinolysis of the embolus in patients with SMA embolism.^20^,^21^ However, while the endovascular approach may rapidly restore the blood flow to the bowel, the time needed for local lysis is variable and the bowel viability cannot be assessed with laparotomy after, if needed.^22^ Also, numerous reports of complications after local thrombolysis were reported, possibly compromising the outcome.^21^,^23^,^24^

Percutaneous mechanical thrombectomy showed good results in our series with dramatic improvement of symptoms present immediately in all patients. No post procedural complications were present in our patients, probably due to absence of any additional pharmacologic lysis.

The main advantages of this technique are rapid and effective removal of large thrombus without the need for local thrombolysis and its minimal invasiveness, thus avoiding the complications associated with surgery. The seriousness of potential vessel damage should not be disregarded and the risk probably increases with smaller vessel diameters. Still, such complications can be easily managed by simple coiling, as performed in one of our patients.

It is of outmost importance that two phases of CTA for bowel ischemia exclusion should be performed prior to the treatment, since SMA recanalisation in patients with necrosed bowel could have disastrous consequences. Also, the location of the embolus should be considered. The recanalisation of completely occluded SMA ostium might be technically challenging and potentially dangerous due to risk of distal arterial embolization; mechanical recanalization of distal branches is difficult and might cause a rupture.

The three patients reported here represent the only patients with occluded AMI, treated with percutaneous mechanical thrombectomy devices at our Institution until now. No permanent complications were observed and good long-term results suggests that Aspirex^®^ and Rotarex^®^ thrombectomy is a good alternative to established surgical treatment in selected cases. Still, by avoiding surgery, the bowel ischemia or necrosis may progress, during the time between CTA and percutaneous procedure, and if the patient’s condition does not improve, laparotomy may follow.
